# Revising acute care systems and processes to improve breastfeeding and maternal postnatal health: a pre and post intervention study in one English maternity unit

**DOI:** 10.1186/1471-2393-12-41

**Published:** 2012-06-06

**Authors:** Debra Bick, Trevor Murrells, Annette Weavers, Val Rose, Julie Wray, Sarah Beake

**Affiliations:** 1 Kings College, London, Florence Nightingale School of Nursing and Midwifery, London, UK; 2 Royal Berkshire NHS Foundation Trust, Reading, UK; 3 The University of Salford, School of Nursing and Midwifery, Manchester, UK

## Abstract

****Background**:**

Most women in the UK give birth in a hospital labour ward, following which they are transferred to a postnatal ward and discharged home within 24 to 48 hours of the birth. Despite policy and guideline recommendations to support planned, effective postnatal care, national surveys of women’s views of maternity care have consistently found in-patient postnatal care, including support for breastfeeding, is poorly rated.

****Methods**:**

Using a Continuous Quality Improvement approach, routine antenatal, intrapartum and postnatal care systems and processes were revised to support implementation of evidence based postnatal practice. To identify if implementation of a multi-faceted QI intervention impacted on outcomes, data on breastfeeding initiation and duration, maternal health and women’s views of care, were collected in a pre and post intervention longitudinal survey. Primary outcomes included initiation, overall duration and duration of exclusive breastfeeding. Secondary outcomes included maternal morbidity, experiences and satisfaction with care. As most outcomes of interest were measured on a nominal scale, these were compared pre and post intervention using logistic regression.

****Results**:**

Data were obtained on 741/1160 (64%) women at 10 days post-birth and 616 (54%) at 3 months post-birth pre-intervention, and 725/1153 (63%) and 575 (50%) respectively post-intervention. Post intervention there were statistically significant differences in the initiation (p = 0.050), duration of any breastfeeding (p = 0.020) and duration of exclusive breastfeeding to 10 days (p = 0.038) and duration of any breastfeeding to three months (p = 0.016). Post intervention, women were less likely to report physical morbidity within the first 10 days of birth, and were more positive about their in-patient care.

****Conclusions**:**

It is possible to improve outcomes of routine in-patient care within current resources through continuous quality improvement.

## **Background**

Each year, the majority of the three-quarters of a million women who have a baby in the United Kingdom (UK) are admitted to hospital for the birth, following which they are transferred to a postnatal ward prior to discharge home. For many women, this will be their first experience as a hospital in-patient [1]. Care on the postnatal ward is led by midwives, supported by maternity care workers who may or may not have undergone a period of training [2]. Despite evidence of widespread and persistent maternal morbidity [3,4], there has been limited revision to the organisation, timing and content of postnatal care which continues to focus on a small number of physical health outcomes, for example vaginal blood loss and assessment of uterine involution. Over the last two decades, in addition to higher patient turnover intervals [5,6] the number of women in the UK who have a caesarean birth has increased [7], outcomes of which are associated with higher risk of severe maternal morbidity relative to vaginal birth [8]. Although caesarean section (CS) is classed as major abdominal surgery, the content of routine postnatal care is unlikely to incorporate specific aspects of post-operative care, with limited guidance on wound management and effective pain management [9,10]. Rates of severe morbidity in general are also increasing [11], with evidence of more complex health needs among women who become pregnant leading to poor pregnancy outcome [12].

Breastfeeding rates in the UK are among the lowest in Europe. Early results from the Infant Feeding Survey 2010 [13] showed that overall initiation rates in the UK increased from 76% in 2005 to 81% in 2010 (for England, rates were 78% and 83% respectively) however, the definition of breastfeeding used in the survey is contentious with data on women who only ever put their baby to the breast on one occasion included [14]. Data on the duration of feeding from the 2010 survey were not available at the time of writing, although the previous survey found a dramatic reduction in the proportion of UK women still exclusively breastfeeding at one week post birth (45%) and less than 1% of women exclusively breastfeeding at six months.

The National Institute for Health and Clinical Excellence (NICE) issued a guideline in 2006 to inform routine postnatal care within the National Health Service (NHS) in England and Wales for women and their babies. Guideline recommendations included the need for individualised care, tailored to the needs of each woman and informed by her pregnancy and birth history, with evidence based recommendations to inform maternal and infant health, infant feeding and the planning and organisation of care. Data from a recent survey of first-time mothers found many women were not receiving care as recommended by NICE [15] with the potential that postnatal care standards in England declined during the last decade [16].

Following an increase in complaints from women about the quality of inpatient postnatal care, including lack of support for breastfeeding, senior clinical managers at one large maternity unit in the south of England aimed to improve the provision of routine postnatal care, supported by implementation of NICE guidance [15]. To address how systems and processes across the organisation could be revised to promote and sustain a ‘seamless’ transfer of women from birth to discharge home and identify barriers and facilitators to enhance postnatal care, a decision was made by the multi professional team (which included the authors) to adapt a model of continuous quality improvement (CQI). The CQI model views quality improvement as an ongoing activity integrated within the organisation and focused on activities determined by those seeking to inform change. It is an approach which emphasises the role of senior management engagement with project teams, and importance of measurement [17], with many CQI interventions reflecting the Plan Do Study Act (PDSA) cycle.

As it became apparent that changes to support evidence based postnatal care needed to be made across the organisation, the importance of engaging as many staff as possible was recognised. Results from a pre and post implementation survey of women at 10 days and 3 months to collate data to compare breast feeding, maternal health outcomes and experiences of care are presented here. The study was funded by The Burdett Trust for Nursing.

## **Methods**

### **Setting**

Around 6,000 women a year give birth at the unit which, when the project commenced in 2007, had a 28% caesarean section rate, a 58% spontaneous vaginal birth rate, and 14% vaginal instrumental birth rate. There were two postnatal wards, each with 26 beds to accommodate women and their infants, staffed by midwives, registered nurses, maternity support workers and nursery nurses. One postnatal ward was part of an integrated midwifery-led unit for low risk women and their babies. The second ward admitted high risk postnatal women and their babies, and women whose babies required transitional care. Capacity issues identified included frequent need to move midwives working on the postnatal wards to cover the labour ward, and a lack of postnatal beds. Midwives were responsible for overseeing all aspects of clinical care on the postnatal wards. Community midwifery teams took over a woman’s care on her discharge home from hospital. Ethics committee approval to undertake the study was obtained via the National Research Ethics Committee (reference number 07/H0505/124).

### **Planning the intervention**

The intervention followed a number of steps informed by CQI. This approach enabled the team to identify where change could be achieved across the organisation to support breastfeeding initiation and duration, enhance women’s postnatal recovery informed by evidence and their views of their inpatient care and preparation for discharge home. Several steps were taken prior to implementing change. Initially, a wide range of stakeholders were interviewed to elicit their perceptions of barriers and facilitators to effective postnatal care in hospital including those of women using the service [18], midwives, obstetricians and senior clinical managers in midwifery. Focus groups were held with midwives from across the acute unit and community teams. Process mapping of the ‘journey’ of postnatal women through the organisation to identify bottle-necks in the system following different modes of birth was carried out by a multi-disciplinary team comprising senior midwives, obstetricians, practice educators and the unit modernisation team. This preliminary work highlighted a number of areas where revisions to care could be introduced, for example, prescriptions for postnatal analgesia could be offered to women attending for pre-operative assessment prior to planned CS birth, rather than waiting for a prescription to be issued at hospital discharge.

It was also important to address how care was documented to comply with the Clinical Negligence Scheme for Trusts (CNST) maternity clinical risk management standards [19]. Work with the management and clinical governance teams took place to review and revise the maternal and neonatal postnatal records in line with CNST requirements. This was also an opportunity to develop records to support the midwife to provide, plan and document the content of care provided in hospital and on transfer home based on enhanced support for breastfeeding and the identification of maternal physical and psychological health needs, informed by evidence [15].

The new record included the introduction of a symptom checklist to prompt early identification and management of common maternal morbidity (for example, breastfeeding issues, backache, urinary problems, perineal pain) and signs and symptoms of potentially severe morbidity (for example, upper genital tract infection and raised blood pressure). Midwives who identified health problems were asked to manage or refer women as appropriate based on their clinical judgement. At the request of the hospital clinical governance team, a Modified Obstetric Early Warning Score (MEWS) tool was included in the new record to enable clinical staff to detect rapid deterioration in maternal physical health. The tool required a score from 0 – 3 to be recorded for each observation included as part of a woman’s inpatient postnatal assessment with respect to urine output, systolic and diastolic blood pressure reading, pulse rate, respiratory rate and conscious level. A MEWS score of 4 or more indicated immediate referral to the medical team. The new, hand held maternal and neonatal record was evaluated as part of the intervention. In line with NICE guidance [15], women were offered the new records from 28 weeks gestation to support the provision of information about plans and needs following the birth of their baby.

### **Content of the intervention**

Following the preparatory development work, a number of changes were implemented over a 10 month period including the piloting and introduction of the new postnatal records. Revisions to routine hospital systems and processes included longer stays on delivery suite from a maximum of two to three hours post vaginal birth to encourage skin-to-skin contact and initiation of breastfeeding. Responsibility for care of women classed as high risk during pregnancy and/or labour was handed from the obstetrician to the midwife immediately following the birth, if appropriate. Postnatal discharge preparation commenced on delivery suite, with midwives asked to complete computer records for women requesting early hospital discharge. Following feedback from women [18], a range of sources of information for parents were introduced onto the wards, including practical infant care demonstrations. An existing postnatal information booklet which contained information about the postnatal ward was also revised and translated into Polish and Urdu. Eighteen half day workshops attended by over 100 clinical staff, mainly midwives and maternity support workers, were held to discuss revisions to care systems and processes, to explain the new postnatal notes and provide an opportunity for discussion.

### **Data collection**

To assess if changes were associated with improved breastfeeding and maternal health outcomes, and enhanced women’s views of care, surveys of women at 10 days and three months post-birth were completed pre and post implementation of the QI intervention package using a specifically developed questionnaires. Primary outcomes included breastfeeding initiation, duration of any breastfeeding and duration of exclusive breastfeeding at 10 days and three months. As one of the aims of revising the postnatal maternal records was to prompt identification of maternal morbidity, including common problems and onset of more severe morbidity, secondary outcomes included maternal physical and psychological health (as measured using the Edinburgh Postnatal Depression Scale (EPDS) [20].

Women were eligible for recruitment if they were aged over 16, able to speak and read English and had not experienced a stillbirth or neonatal death. All women on the postnatal ward who were eligible to participate were offered an information leaflet about the study, a copy of the 10 day questionnaire on the postnatal ward by the research midwife or when she was not there by the ward midwives. The women were asked to return the completed questionnaires to the study team using a pre-paid envelope, the return of the questionnaire viewed as the women giving consent to participate. Questionnaires at three months were mailed to all women who had returned a 10 day questionnaires. Recruitment and follow up to the pre-intervention study took place from January – June 2008 inclusively, and post-intervention from April – September 2009 inclusively, which enabled a three month period of recruitment and a three month period of follow up of those who consented. To detect a difference in the initiation of breastfeeding from 85% to 87%, a 2% increase in breastfeeding initiation per year in line with Department of Health guidance [21], with 80% confidence at a level of p < 0.05, a minimum of 1073 women would need to be recruited.

### **Statistical analyses**

Nearly all outcomes of interest were measured on a nominal scale. These were compared pre and post intervention using logistic regression (Yes/No). Time in days before breastfeeding stopped was compared pre and post intervention using Cox regression, and scores on the EPDS were compared using analysis of covariance. Covariates were included in models to account for variation in individual characteristics between women. All regression models included age, parity, ethnicity, mode of birth, length of inpatient stay, number of midwifery contacts and number of midwives seen at home (from the questionnaire at 10 days) as covariates. Additional covariates that indicated how much help mothers received with breast feeding at hospital and home were included when modelling the dependent variables ‘ever’ breast fed and still breast feeding at 10 days.

Analysis tables show either the odds ratio, hazard ratio or mean difference (β) with 95% confidence intervals (95% CI) that statistically compare pre and post-intervention outcomes adjusting for the covariates listed above. On one occasion the model did not converge because the condition (mastitis) was rare therefore it was not possible to present the 95% CI. The p value in this case comes from a Fisher’s Exact test. A test statistic attained statistical significance if p ≤ .05.

## **Results**

Of 1160 women recruited pre intervention, 741 (64%) returned a completed questionnaire at 10 days and 616 (54%) returned a completed questionnaire at three months. Post intervention, 725 (63%) of 1153 women recruited returned a 10 day questionnaire and 575 (50%) returned a three month questionnaire. There were no differences between the two time periods and maternal characteristics (Table 1) and no differences in maternal or obstetric characteristics of women who were not recruited during the study period compared with those who were. The majority of women were white European, including a large number of women from Eastern Europe with Polish the second most common language spoken by women. There was a significant increase in the number of births by caesarean section pre and post intervention, with a concurrent decrease in instrumental births.

**Table 1:** Baseline maternal, obstetric and service characteristics

**Maternal, obstetric and service characteristics**	**Pre intervention (N = 741)**	**Post intervention (N = 725)**
Age	30.51 (SD 5.62)	30.70 (SD 5.46)
Parity	1.66 (SD .85)	1.67 (SD .86)
White European	603 (81.3%)	603 (83.2%)
Mode of birth		
Spontaneous vaginal	390 (52.6%)	382 (52.7%)
Instrumental	141 (19.0%)	90 (12.4%)
Caesarean section	209 (28.2%)	241 (33.2%)
Length of Stay (days)	2.24 (SD 5.18)	2.40 (SD 5.22)
Number of midwifery contacts at home (at 10 days)	2.84 (SD 1.29)	2.84 (SD 1.18)
Number of midwives seen at home (at 10 days)		
1	153 (20.6%)	135 (18.6%)
2	266 (35.9%)	298 (41.1%)
3	214 (28.9%)	184 (25.4%)
4	85 (11.5%)	57 (7.9%)
4 or more	None	16 (2.2%)
Help available for breastfeeding in hospital		
Yes I was able to get all the help I needed, when I needed it	358 (48.3%)	353 (48.7%)
I got some help sometimes, when needed, but not others	145 (19.6%)	148 (20.4%)
I did not get the help I needed	58 (7.8%)	57 (7.9%)
I didn’t really need help	144 (19.4%)	154 (21.2%)
Not answered	36 (6.7%)	13 (1.8%)
Help available for breastfeeding at home		
Yes I was able to get all the help I needed, when I needed it	311 (42.0%)	300 (41.4%)
I got some help sometimes, when needed, but not others	101 (13.6%)	83 (11.4%)
I did not get the help I needed	34 (4.6%)	37 (5.1%)
I didn’t really need help	201 (27.1%)	207 (28.6%)
Not answered	94 (12.7%)	98 (13.5%)

### **Impact on breastfeeding outcomes**

There were statistically significant differences in breastfeeding outcomes (Table 2). Women post intervention were significantly more likely to still be breastfeeding at 10 days (p = 0.020) and to be breastfeeding exclusively at 10 days post-birth rather than breast and artificially feeding their infants (p = 0.038). The finding for initiating breastfeeding was on the cusp of statistical significance (p = 0.050) favouring mothers post-intervention.

**Table 2:** Breastfeeding initiation, duration and duration of exclusive breastfeeding up to three months post birth

**10 – 12 days**	**Pre intervention (N = 741)**	**Post intervention (N = 725)**	**(95% CI)†**	***p*value (adjusted)**
Ever breast fed	636 (86.1%)	628 (87.4%)	1.439 (1.000, 2.071)	0.050
Still breastfeeding at 10 days	526 (83%)	550 (87.3%)	1.514 (1.069, 2.144)	0.020
Breastfeeding exclusively at 10 days‡	344 (65.8%)	378 (70.3%)	1.339 (1.016, 1.766)	0.038
3 months	Pre intervention	Post intervention (N = 529)		
	(N = 606)			
Breastfed/given expressed breast milk after 10 days (up to 3 months)	466 (76.9%)	448 (84.7%)	1.541 (1.085, 2.189)	0.016
Still breastfeeding at 3 months	328 (70.5%)	319 (71.4%)	1.029 (0.739, 1.433)	NS
Exclusively breastfeeding at 3 months	208 (64.2%)	213 (66.8%)	1.084 (0.742, 1.585)	NS
When did you stop breastfeeding?	Kaplan-Meier mean 57.4 days	Kaplan-Meier mean 62.3 days	0.830 (0.697, 0.989)ǂ	0.037

† odds ratio unless otherwise stated ǂhazard ratio; ‡ as a proportion of women breast feeding exclusively and breastfeeding plus artificial milk.

There were also significant differences on breastfeeding outcomes after 10 days and up to three months post birth. Women post intervention were statistically significantly more likely to have continued to breastfeed within this period of time (p = 0.016), although there was no difference between the groups and still breastfeeding at three months. The hazard ratio from the Cox regression analysis showed that women stopped breastfeeding significantly earlier in the pre intervention group (57.0 days compared with 62.1 days, p = 0.039).

### **Maternal physical and psychological health outcomes**

#### **In hospital**

There was a statistically significant difference in the number of women who reported vaginal blood loss which was heavy or had an offensive odour in hospital, with fewer women post intervention reporting this (p = 0.001, Table 3). Backache was also less likely to be reported (p = 0.076). Although statistical significance was not achieved, fewer women reported the health problems of interest post intervention other than headache. There were no statistically significant differences between the two groups in women’s views of need for emotional support in hospital (139/18.9% pre intervention, 111/16.1% post-intervention). Of those women who reported that they did need emotional support in hospital, there was no difference in being able to speak to a midwife about this (76/54.7% pre intervention, 60/54.1% post intervention).

**Table 3:** Maternal health problems experienced in hospital

**Health problem**	**Pre intervention (N = 741)**	**Post intervention (N = 725)**	**(95% CI) †**	***p*value (adjusted)**
Fever/high temperature	116 (15.7)	88 (12.1)	0.789 (0.576, 1.082)	NS
Excessive vaginal bleeding	96 (13)	71 (9.8)	0.808 (0.572, 1.143)	NS
Offensive vaginal loss	28 (3.8)	5 (0.7)	0.187 (0.071, 0.492)	0.001
Severe or persistent headache	27 (3.6)	29 (4.0)	1.156 (0.647, 2.066)	NS
Perineal pain	172 (23.2)	133 (18.3)	0.887 (0.662, 1.187)	NS
Haemorrhoids	90 (12.1)	81 (11.2)	0.933 (0.665, 1.308)	NS
Constipation	148 (20.0)	130 (17.9)	0.818 (0.608, 1.102)	NS
Difficulty passing urine	41 (5.5)	37 (5.1)	1.039 (0.644, 1.677)	NS
Caesarean wound problems	23 (3.1)	22 (3.0)	0.908 (0.460, 1.793)	NS
Backache	135 (18.2)	104 (14.3)	0.766 (0.571, 1.028)	0.076
Mastitis	2 (0.3)	0 (0.0)	not availableǂ	NS

† odds ratio; ǂ model did not converge successfully, p value taken from Fisher's Exact Test.

#### **At home up to first 10 days**

When asked about health problems experienced following hospital discharge within the first 10 days, there were again statistically significant differences pre and post intervention. Post intervention women were significantly less likely to report offensive vaginal loss, difficulties with passing urine, constipation and mastitis compared with women surveyed pre intervention (Table 4).

**Table 4:** Maternal health problems experienced at home within 10 days of birth

**Health problem**	**Pre intervention (N = 741)**	**Post intervention (N = 725)**	**(95% CI) †**	***p*value (adjusted)**
Fever/high temperature	90 (12.1%)	72 (9.9%)	0.806 (0.571, 1.140)	NS
Excessive vaginal bleeding	53 (7.2%)	46 (6.3%)	0.871 (0.566, 1.341)	NS
Offensive vaginal loss	25 (3.4%)	10 (1.4%)	0.369 (0.169, 0.807)	0.012
Severe or persistent headache	81 (10.9%)	78 (10.8%)	0.990 (0.699, 1.401)	NS
Perineal pain	215 (29.0%)	188 (25.9%)	1.091 (0.829, 1.436)	NS
Haemorrhoids	164 (22.1%)	150 (20.7%)	0.994 (0.761, 1.298)	NS
Constipation	232 (31.3%)	185 (25.5%)	0.719 (0.560, 0.925)	0.010
Difficulty passing urine	45 (6.1%)	26 (3.6%)	0.580 (0.342, 0.986)	0.044
Caesarean wound problems	46 (22.0%)	49 (20.3%)	0.983 (0.612, 1.579)	NS
Backache	251 (33.9%)	217 (29.9%)	0.860 (0.682, 1.085)	NS
Mastitis	49 (6.6%)	29 (4.0%)	0.605 (0.369, 0.990)	0.046

† odds ratio.

#### **At home after 10 days and within 3 months**

At three months, women were asked about their experiences of the same health problems and also asked to complete the EPDS. Women post-intervention were less likely to report constipation, but more likely to report headaches (Table 5). There were no differences in mean EPDS scores or proportion of women who had an EPDS score of ≥13 indicating that they were likely to be suffering from depression (69/11.3% pre intervention and 68/12.8% post intervention), reporting of need for emotional support during this time (178/29.8% compared with 168/31.7%) or experiences of difficulty talking to a health professional about this (98/55.1% compared with 95/56.5%).

**Table 5:** Maternal health problems after 10 days and within 3 months of the birth

**After 10 days & within 3 months**	**Pre intervention N = 616**	**Post intervention N = 575**	**(95% CI) †**	***p*value (adjusted)**
Fever/high temperature	92 (14.9%)	77 (13.4%)	0.850 (0.582, 1.239)	NS
Excessive vaginal bleeding	54 (8.8%)	44 (7.7%)	0.823 (0.503, 1.347)	NS
Offensive vaginal loss	16 (2.6%)	25 (4.3%)	1.301 (0.626, 2.703)	NS
Severe or persistent headache	29 (4.7%)	46 (8.0%)	1.940 (1.109, 3.392)	0.020
Perineal pain	101 (16.4%)	72 (12.5%)	0.938 (0.623, 1.413)	NS
Haemorrhoids	164 (26.6%)	152 (26.4%)	1.024 (0.763, 1.374)	NS
Constipation	163 (26.5%)	127 (22.1%)	0.732 (0.535, 1.002)	0.052
Difficulty passing urine	10 (2.6%)	9 (1.6%)	0.781 (0.306, 1.997)	NS
Caesarean wound problems	61 (9.9%)	56 (9.7%)	0.679 (0.417, 1.107)	NS
Backache	176 (28.6%)	170 (29.6%)	1.010 (0.756, 1.350)	NS
Mastitis	77 (12.5%)	62 (10.8%)	0.706 (0.467, 1.068)	NS
EPDS mean score	6.77 (SD 4.53)	6.74 (SD 4.80)	−0.152 (−0.770, 0.466)ǂ	NS
EPDS score ≥ 13	69 (11.3%)	68 (12.8%)	1.291 (0.850, 1.962)	NS

† odds ratio unless otherwise stated ǂmean difference.

### **Experiences of care**

Women pre intervention were significantly less satisfied (738/77.4% vs. 719/82.1%, p = 0.019) with the overall postnatal care they received for themselves, with no difference in overall satisfaction for the care offered to their babies pre and post intervention. Women pre intervention were significantly less likely to report that care in hospital was better than they had expected (247/33.7% vs. 288/40.2% , p = 0.024).

### **Planning of care**

Pre intervention women were significantly less likely to have had their length of in-patient stay planned with them (374/51.7% vs. 441/61.3%, p < 0.001) and plans for their discharge home discussed with them (415/70.1% vs. 401/77.0% , p = 0.010).

## **Discussion**

Using a CQI approach to identify where change could be introduced into routine systems and processes, it was possible to improve breastfeeding initiation and duration, some maternal physical health outcomes and enhance women’s views of their inpatient care. This is the first study to have addressed how postnatal services in an acute UK maternity setting could be revised in line with evidence.

Improving breastfeeding initiation and the duration of exclusive breastfeeding are important public health priorities for the UK [22], given shorter and longer-term health benefits for women and their infants [23-25]. It was reassuring that the QI initiative impacted positively on breastfeeding. At the time of the study, the unit was not Baby Friendly accredited or working to obtain a certificate of commitment. A recent systematic review of structured compared with non structured programmes to support the initiation and duration of breastfeeding found that although most included studies reported statistically significant increases in breastfeeding initiation in hospital settings, studies were methodologically poor, effect sizes differed and not all found statistically significant differences in breastfeeding duration [26].

There are a number of reasons to support why our multi-faceted intervention made a difference, including an approach which supported ‘doing the right thing at the right time’. An important factor was recognition of the need to target the content and timing of care and provision of information offered as a continuum across pregnancy, birth and the postnatal period. Support for breastfeeding commenced with antenatal information, with subsequent clinical care and processes to support breastfeeding promoted immediately following birth, with midwives on the labour ward asked not to transfer women for at least two hours following the birth to enable women to have longer ‘quiet’ time with their babies to initiate skin to skin care. The 2005 UK Infant Feeding Survey found breast feeding initiation higher for babies exposed to early skin to skin contact (79% immediately and 87% within an hour, compared with 57% of babies with no such contact) and initial incidence of skin to skin contact correlated with breastfeeding prevalence at one and two weeks post birth [14]. In line with NICE guidance [15] the new postnatal record prompted the early identification and management of breastfeeding issues as part of in-patient care. A combination of these factors could have impacted on women’s confidence to breastfeed.

Mode of birth was adjusted for within the analysis, although it was of note that there was a large increase in CS births pre and post intervention. CS births can negatively impact on breastfeeding initiation, with evidence that women who have had operative birth are less likely to commence breastfeeding [27,28], but are no more likely to discontinue breastfeeding than women who had a vaginal birth [14,15].

The NICE postnatal care guideline [15] recommended that all maternity care providers in England and Wales should implement an externally evaluated structured programme that encourages breastfeeding, using the UK Baby Friendly Hospital Initiative as a minimum standard. Currently, evidence of which type of structured support is more likely to increase the initiation and duration of breastfeeding in England and Wales is unclear. Although more women were exclusively breastfeeding at three months post intervention, this was not statistically significant. Nevertheless the revisions to care reflected ‘best practice’ within routine service provision and could be a useful approach to adopt elsewhere given the increased demands on finite health service resources.

The inclusion of a symptom checklist in the new postnatal records appeared to impact on early identification of physical health outcomes, which could then be managed by the midwife or referred as appropriate. Women post-intervention were less likely to report offensive vaginal loss within the first 10 days of the birth, an important indicator of potential serious maternal infection [15]. Use of Modified Obstetric Early Warning Scores (MEWS) have been recommended by CMACE [12,29] to detect early onset of severe maternal morbidity, although evidence to support the effectiveness of these tools in obstetric populations is not currently available. Data on use of the MEWS were not evaluated as part of this study, and it is not possible to assess if use prompted identification of potentially serious morbidity. As the MEWS tool in use did not include ‘triggers’ of specific signs and symptoms of severe postnatal morbidity, for example, temperature, lower abdominal pain or increased vaginal blood loss, it is more likely that use of the symptom checklist prompted identification. As other significant differences in reported physical morbidity, including backache (in hospital), constipation, difficulty passing urine and mastitis (within first 10 days at home) were also found post-intervention, would confirm this.

At three months, it is less clear that the QI intervention impacted on longer-term physical health, with evidence that resolution of physical health problems, even with directed community based midwifery interventions and longer-term follow up, may be difficult to achieve. MacArthur et al. [30,31] conducted a large cluster randomised trial of a new model of midwifery-led postnatal community care, which extended midwifery contact to 28 days, with a final midwife contact at 10–12 weeks which replaced the six – eight week appointment with the family doctor. Primary trial outcomes at 4 and 12 months post-birth included physical and mental health and well-being as assessed using the Physical and Mental Health Components scores of the SF36 (PCS and MCS) and the EPDS. There were no differences in PCS scores at 4 and 12 months post-birth. Secondary trial outcomes included physical morbidities at 12 months post-birth, with no statistically significant differences in outcomes other than fatigue and haemorrhoids. In the current study most women would have been discharged by their midwife at around 10 – 14 days post birth, with no further follow up of their maternity care until the 6 – 8 week appointment with the family doctor. Our findings suggest that some symptoms are amenable to early midwifery intervention, with a need to promote ways in which longer-term physical morbidity can be addressed.

Unlike the current study, MacArthur et al. [30,31] found significant differences in maternal mental health outcomes at 4 and 12 months post-birth following their community based intervention. We found no difference in EPDS scores at 3 months post-birth and no difference in emotional health needs in hospital, an area previously reported as a neglected aspect of women’s postnatal care [16,32]. It is possible that the women in the current study did not consider emotional health a priority when a hospital in-patient, that in the short space of time prior to discharge they did not consider their feelings, or care to identify these outcomes was not sufficiently different pre and post intervention. Mental health disorders among women following birth are an important area for consideration, and the need to review strategies to prompt early identification and management is recognised [33,34]. The trial by MacArthur et al. [30,31] remains the only universal intervention to have impacted on maternal mental health outcomes and highlights the potential benefit of planned, effective midwifery care.

An earlier study from Melbourne, Australia [1] which compared women’s views pre and post intervention of a number of initiatives across a network of four public maternity hospitals, also attempted to improve outcomes of routine in-patient postnatal care. The intervention package included discussion visits with midwives in the third trimester of pregnancy, written information for service users, rotation of staff across intra-partum and postnatal clinical areas and introduction of evidence based guidelines and protocols. A four year evaluation based on outcomes from three units included postal surveys at three months post-birth of women at baseline (1922/1256, 65.3%) and post-implementation (1050/1829, 57.4%). Post-implementation there were statistically significant improvements in overall ratings of hospital postnatal care and level of advice offered. There were no differences in morbidity outcomes, but fewer women post intervention required help and advice on their own health at home suggesting strategies had benefited women’s health.

As part of our work, we asked women what information, support and advice they needed to better prepare them for going home [18]. Women were more positive about their experience, satisfaction with and planning of care post intervention, emphasising that service providers considering revisions to routine care should reflect what users consider important if positive feedback is to be achieved, including provision of practical demonstrations of infant care on the wards. There has been a major policy shift in the NHS to engage service users in decisions about their care [35] and a move for greater involvement of patients, carers and clinicians in setting priorities for research in areas where treatment uncertainties exist [36]. Service user engagement in planned service revision is clearly important for maternity service development within a QI initiative as women may highlight areas for change which clinicians and managers may not necessarily have deemed a priority. Midwives viewed overall changes as positive for the women and for their practice, although there was some concern that the new postnatal records took longer to complete as they were more comprehensive than the previous notes used at the study site [37].

The limitations of our study have to be considered, as well as the strengths. It was disappointing that there was some loss to follow up of women at three months despite sending two reminder questionnaires, however our response rates are comparable with similar postnatal studies [1]. The study design within a CQI framework meant that any potential confounding factors could not be controlled for, and influences on outcomes as a result of other changes across the organisation could not be accounted for. Data to inform economic analyses were also not planned as part of this work, although no additional resources were requested to implement the CQI intervention. That there could have been ‘knock-on’ effects from some of the revisions to care (for example, asking women to be kept on the labour ward for a longer period of time) cannot be discounted.

The study was not designed to specifically compare the occurrence of maternal health problems pre and post-intervention. A much larger sample would be required for this purpose. Nevertheless, that we had a positive impact on a number of important outcomes particularly when current staffing levels and skill mix within NHS maternity services are of concern [38] demonstrates that a CQI approach to revise routine systems and processes could support improved outcomes of routine care in other UK maternity units.

## **Conclusions**

Achieving sustained change in an area of the maternity service where women may only be admitted for a few hours post-birth is an on-going challenge. Using a CQI approach provided an excellent opportunity to engage all relevant stakeholders, including women, to consider and identify where systems and processes could be revised within current resources to improve the quality and outcomes of in-patient care following birth. The improvement initiative was developed in response to women’s views of care and recognition of an ‘evidence to practice’ gap. Work to identify the issues, how these could be resolved and how best to implement and sustain change were important first steps before adopting a CQI approach to address the ‘knowing-doing’ gap [39]. We have shown that CQI can support implementation of evidence to enhance breastfeeding outcomes, reduce maternal morbidity and enhance women’s views of in-patient postnatal care and would encourage other maternity providers to consider a similar approach.

## **Competing interests**

The authors declare that they have no competing interests.

## **Authors' contributions**

DB conceived the study, was lead investigator, participated in its design and data analysis and drafted the manuscript. TN performed the statistical analysis and contributed to the manuscript. SB participated in the data analysis and coordination of the study and contributed to the manuscript. VR collected the data and contributed to the manuscript. AW and JW contributed to the design of the study and the manuscript. All authors read and approved the final manuscript.

## Pre-publication history

The pre-publication history for this paper can be accessed here:

http://www.biomedcentral.com/1471-2393/12/41/prepub
